# How Does Complement Affect Hematological Malignancies: From Basic Mechanisms to Clinical Application

**DOI:** 10.3389/fimmu.2020.593610

**Published:** 2020-10-29

**Authors:** Shanshan Luo, Moran Wang, Huafang Wang, Desheng Hu, Peter F. Zipfel, Yu Hu

**Affiliations:** ^1^ Institute of Hematology, Union Hospital, Tongji Medical College, Huazhong University of Science and Technology, Wuhan, China; ^2^ Department of Integrated Traditional Chinese and Western Medicine, Union Hospital, Tongji Medical College, Huazhong University of Science and Technology, Wuhan, China; ^3^ Department of Infection Biology, Leibniz Institute for Natural Product Research and Infection Biology, Hans Knöll Institute, Jena, Germany; ^4^ Faculty of Biological Sciences, Friedrich Schiller University, Jena, Germany

**Keywords:** complement, hematological malignancies, immune evasion, tumor progression, immunotherapy

## Abstract

Complement, as a central immune surveillance system, can be activated within seconds upon stimulation, thereby displaying multiple immune effector functions. However, in pathologic scenarios (like in tumor progression), activated complement can both display protective effects to control tumor development and passively promotes the tumor growth. Clinical investigations show that patients with several hematological malignancies often display abnormal level of specific complement components, which in turn modulates complement activation or deregulated cascade. In the past decades, complement-dependent cytotoxicity and complement-dependent cell-mediated phagocytosis were fully approved to display vital roles in monoclonal antibody-based immunotherapies, especially in therapies against hematological malignancies. However, tumor-mediated complement evasion presents a big challenge for such a therapy. This review aims to provide an integrative overview on the roles of the complement in tumor promotion, highlights complement mediated effects on antibody-based immunotherapy against distinct hematological tumors, hopefully provides a theoretical basis for the development of complement-based cancer targeted therapies.

## Introduction

Complement was initially identified more than 100 years ago due to a result of its bactericidal activity ‘complementary’ to the action of immunoglobulins and the role in phagocytosis of cellular debris (1, 2). As an essential part of innate immunity and an evolutionary old system, complement is highly conserved among a wide variety of species, emphasizing its importance in immune defense throughout evolution (3). Complement-dependent cytotoxicity (CDC) and complement-dependent cell-mediated phagocytosis (CDCP) were fully approved to display vital roles in maintaining homeostasis, fighting against infection and even in monoclonal antibody-based immunotherapies, especially in therapies against hematological malignancies. Dysfunction of each step of the whole cascade disrupts homeostasis and ultimately leads to severe diseases, such as tissue damage, autoimmune diseases, infection and tumor progression (4). Clinical investigations show that patients with hematological malignancies often display abnormal level of complement components, which in turn modulates complement activation.

Hematologic malignancies are complex groups of disorders, which becomes more and more into people’s focus since they are often diagnosed in clinic. Although the general cure rate of hematologic malignancies has been greatly improved and some types have even high cure rates now, great challenges still exist due to the large number of disease subtypes and high heterogeneity. According to the 2018 global cancer statistics, the incidence and mortality of non-Hodgkin lymphoma (NHL) ranks first in hematological neoplasms, followed by leukemia, multiple myeloma (MM), and Hodgkin lymphoma (HL) (5). The causes of the diseases are various, but the host immune conditions and tumor virulence factors are the main determinants which closely correlated with the disease state. The management of hematologic malignancies has traditionally relied on chemotherapy and/or radiotherapy regimens to control the tumor progression and prolong the life span (6). However, over the past two decades, significant progress has been made for the development of monoclonal antibody (mAb) therapies for hematologic malignancies. For example, anti-CD20 mAbs, mainly rituximab (RTX) or obinutuzumab, combined with chemotherapy agents have been approved for treatment of diffuse large B-cell lymphoma (DLBCL), follicular lymphoma (FL) and chronic lymphocytic leukemia (CLL), respectively (7, 8). Complement-dependent cytotoxicity and complement-dependent cell-mediated phagocytosis display vital roles in mAb-based immunotherapies. However, tumor-mediated complement evasion might be a big challenge for such a therapy.

In this review, we will first summarize complement activation, function, and regulation, present an overview of the “double edged” roles of complement in tumor progression, then provide a deep insight into the roles of complement in hematological malignancies, and further discuss complement mediated effects on antibody-based immunotherapy against hematological tumors.

## Complement Activation, Function and Regulation

**Figure 1:**
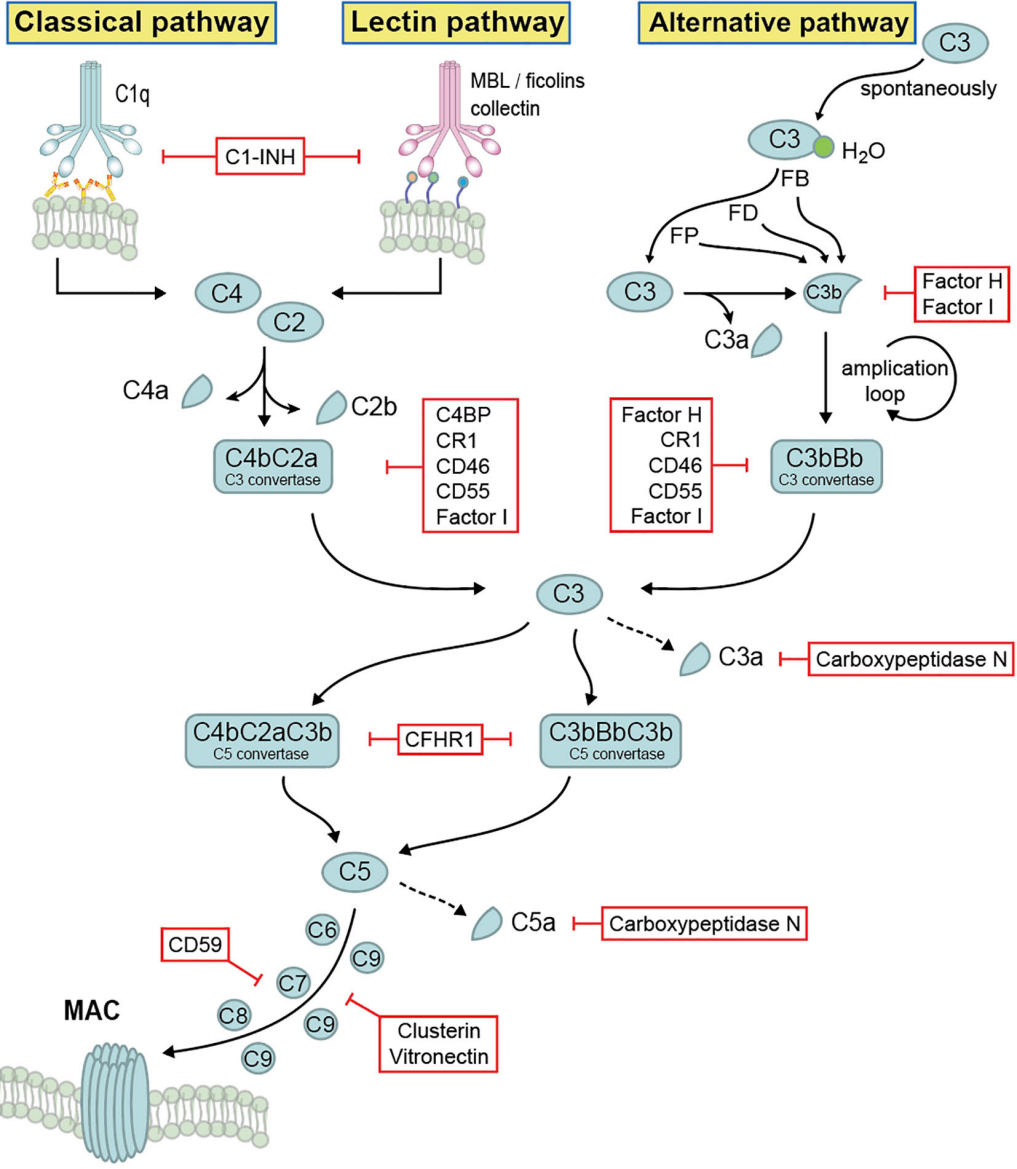
Complement activation, effector function and regulation. Complement system is activated by three different pathways, then merged at the level of C3 cleavage, followed by C5 convertase formation and generation of terminal complement complex. Upon activation, different activation products are generated, which display multiple immune effector functions. The whole system is tightly self-controlled by different regulators.

The complement system comprises more than 60 different components which include main components, various activation products, effector components, regulators, and several surface bound complement receptors. Soluble complement exists in the body fluids, displaying multiple immune effector functions. Complement activation occurs in a sequential manner *via* three different pathways and consists of four main steps: initiation, C3 convertase formation and amplification, C5 convertase formation, and the assembly of the terminal complement complex (TCC), also known as membrane attack complex (MAC). The alternative pathway (AP) is initiated spontaneously and constantly. The lectin pathway (LP) is activated upon binding of mannose-binding lectin to mannan and carbohydrate structures on microbial surfaces. The classical pathway (CP) is activated *via* antigen–antibody complexes or by C-reactive protein (4, 9). Activation of all three pathways results in the generation of C3 convertases that cleave C3 into C3a and C3b, followed by C5 convertase formation that cleaves C5 into C5a and C5b, and the generation of TCC (3, 4) (**Figure 1**).

The cleavage product C3b binds to target surfaces, where it acts as opsonin and mediates recognition and phagocytosis by host immune effector cells (10, 11). C3a and C5a function as anaphylatoxins which initiate inflammation. Furthermore, C3a also has antimicrobial activity by binding to the cell surface of microbes and induces membrane perturbations and release of extracellular material (12). In addition, complement also functions as a link between innate and adaptive immunity. C3 synthesis by myeloid cells, a relatively minor source of complement, provides a critical function during the induction of humoral B responses to peripheral herpes simplex virus infection. Further, macrophages derived from bone marrow produce sufficient C4 to restore the humoral response to virus infection in C4-deficient animals, demonstrating local complement C3 and C4 production are required to enable efficient B cell responses (13, 14). Immune responses of T cell to *Listeria monocytogens* were impaired in the absence of C3 (15). Besides that, complement activation products C5a/C3a and its receptors (C5aR/C3aR) have a clear role in directly and indirectly promoting T cell activation and proliferation and as such, promoting allograft rejection, autoimmunity, and fighting infection (16–18).

Due to its potency and the damaging effects, many complement components are engaged in regulation (**Figure 1**). Complement regulators function at all levels of the cascade and are classified into two major classes: fluid phase regulators and membrane-integral complement receptors, such as Factor H, Factor H like protein 1 (FHL-1), C4b binding protein (C4BP), C1 inhibitor (C1INH), complement Factor H related protein 1 (CFHR1), CFHR2, CFHR3, CFHR4, CFHR5, as well as complement receptor 1 (CR1), CR2, CR3, CR4, CD46, CD55, CD59, CRIg, vitronectin, clusterin as well as carboxypeptidase N (2, 19, 20). Being highly regulated by these regulators, complement forms an important, central immune defense line and mediates cell integrity and tissue homeostasis. Additionally, beyond complement regulation, several of the above-mentioned regulators have additional activities, such as mediating cell adhesion and extracellular matrix interaction, or linking the complement cascade with other important physiological networks (*e.g.* the coagulation cascade) (21). However, due to the complement dysfunction, many disease pathologies including tumor progression, tissue damage, autoimmune diseases and infection may take place (22).

## Role of Complement in Tumor Progression: The Two Sides of the Coin

### Complement Mediated Anti-Tumor Effects

The complement system is a double-edged sword in tumor development since complement activation is not only involved in anti-tumor cytotoxicity and immune responses, but also promotes tumor development directly and indirectly. Regarding to its anti-tumor side, complement, upon activation, displays various controlling effects (*e.g.* C3b/iC3b mediated phagocytosis and TCC mediated cell lysis) on various tumor cells including both solid tumors and hematological malignancies (23). For example, upon treatment of CLL, complement activation was initiated by anti-CD20 monoclonal antibody RTX, thereby displaying efficient CDC and CDCP to clear the tumor cells (24). However, in order to block the toxic effects of activated complement, and to survive, tumor cells, similar to infectious microbes utilize multiple evasion strategies to actively escape complement attack and immune surveillance. For example, lung, ovarian, glial and hematological tumor cells show enhanced expression and surface binding of soluble regulators including Factor H, FHL-1, FHR1, FHR-4, FHR5, and C4BP. These up-regulated/bound complement regulators further display the cofactor activity, which function together with factor I to block complement activation at the level of C3 convertase, thereby leading to complement evasion (25–29). Similarly, the membrane-bound complement inhibitors (*e.g.* CD46, CD55, and CD59) are up-regulated in various primary tumors and tumor lines to evade the complement attack (30, 31). Serglycin is another endogenous complement inhibitor secreted by human MM cell lines. Serglycin can inhibit both the classical and lectin pathways’ activation by direct interaction with C1q and mannose-binding lectin, thereby blocking complement mediated immune effector effects on MM cells (32).

### Complement Mediated Promotion of Tumor Growth

Besides the controlling effect, over-activation of complement also promotes tumor growth through the pro-inflammatory properties of effector compounds, which is in line with established cancer-promoting effects of chronic infections (33). Chou et al. reported that local production and activation of complement effector compounds were distinctly important for promoting tumor growth, while the systemic production of complement by liver did not affect cancer (34).

### C3a–C3aR and C5a–C5aR Mediated Proliferative Effects

The proliferative effect on tumors is directly correlated to the activation of complement cascade, as derived by C3a and C5a *via* C3aR and C5aR1 mediated PI3K/AKT signaling pathway (34). Shu et al. reported that C3a-C3aR signaling *via* PI3K/AKT pathway in carcinoma associated fibroblasts facilitated the metastasis of breast cancer. Targeting C3aR signaling was shown to be a potential anti-metastasis strategy for breast cancer therapy (35). Upon signaling by C5a stimulation, C5aR1 expressed tumor cells underwent cytoskeletal rearrangement (*e.g.* filopodia formation, membrane ruffling) and furthermore released matrix metalloproteinase, which lead to increased tumor cell motility and invasiveness both *in vitro* and *in vivo* (36). Another report showed that C5aR1 signaling induced myeloid-derived suppressor cells to produce larger amounts of reactive oxygen species and reactive nitrogen species, which inhibited CD8+ T cell mediated anti-tumor activity, thereby leading to tumor growth (37). Vadrevu et al. showed that in a lung cancer metastatic model, C5aR1 blockage resulted in decreased lung metastasis due to reduced TGF-b and IL-10 production by myeloid-derived suppressor cells since both TGF-b and IL-10 induced regulatory T-cell generation and facilitated an immunosuppressive Th2-based T cell responses (38). Further *in vivo* analysis showed that C5aR1 signaling promotes melanoma growth by promoting infiltration of immunosuppressive leukocyte populations into the tumor microenvironment, whereas C5aR2 has a more restricted but beneficial role in limiting tumor growth, further proving the “double-edged” role of complement activation in tumor promotion (39).

### Other Complement Components Mediated Effects

Besides the complement receptors mediated effects, different components of the main cascade also display onco-progression ability, such as C1q, C3 and C5b-9. Bioinformatics analysis by Mangogna et al. showed that high levels of C1q have a favorable prognostic index in basal-like breast cancer for disease-free survival, and in HER2-positive breast cancer for overall survival (40). C1q acts in the tumor microenvironment as a cancer-promoting factor in a complement dependent as well as independent manner. In clear-cell renal cell carcinoma, tumor associated macrophages produce high densities of C1q, which together with tumor cell expressed C1r, C1s, C4, and C3 initiates CP activation. The activation products as well as tumor associated macrophages-derived C1q further promotes an immunosuppressed microenvironment characterized by high expression of immune checkpoints (*e.g.* programmed cell death protein (PD)-1, Lag-3, programmed cell death ligand 1 (PD-L1), and PD-L2), thereby fueling tumor progression. Mice deficient in C1q, C3, and C4 displayed decreased tumor growth (41).

Yuan et al. reported that local complement C3 overexpression activated JAK2/STAT3 pathway and promoted gastric cancer progression. Further clinical investigation showed that C3 and C3a expression was markedly enhanced in gastric cancer tissues at both mRNA and protein levels compared with those in paired non-tumorous tissues. High C3 deposition was identified as an independent prognostic factor of poor 5-year overall survival, suggesting that local C3 deposition in the tumor microenvironment is a relevant immune signature for predicting prognosis of gastric cancer (42). *In vivo* data showed that the knockdown of C3 suppressed hepatic stellate cells-promoted hepatocellular carcinoma development (43).

Further, sublytic C5b-9 displays tumor-promoting properties by activating signal transduction pathways (*e.g.*, Gi protein/PI3K/Akt kinase and Ras/Raf1/ERK1) and modulating the activation of cancer-related transcription factors, while shielding malignant cells from apoptosis (44). Such complement promoted tumor progression also actively involved in cutaneous squamous cell carcinoma, which was nicely reviewed by Pilvi Riihilä et al. (45).

Based on these roles of complement in promoting cancer progression, more and more complement proteins are becoming potential candidates for cancer targeted therapy and numerous new anti-complement drugs are under clinical development (46). For example, C3aR and C5aR are recently classified as a new class of immune checkpoint receptor in cancer immunotherapy (47). In addition, humanized soluble CR1-Fc fusion protein were generated to target C3b/C4b and its therapeutic effect was conferred in a colitis-associated colorectal cancer model and orthotopic 4T1 breast cancer model (48).

## Complement in Hematological Malignancies

**Figure 2:**
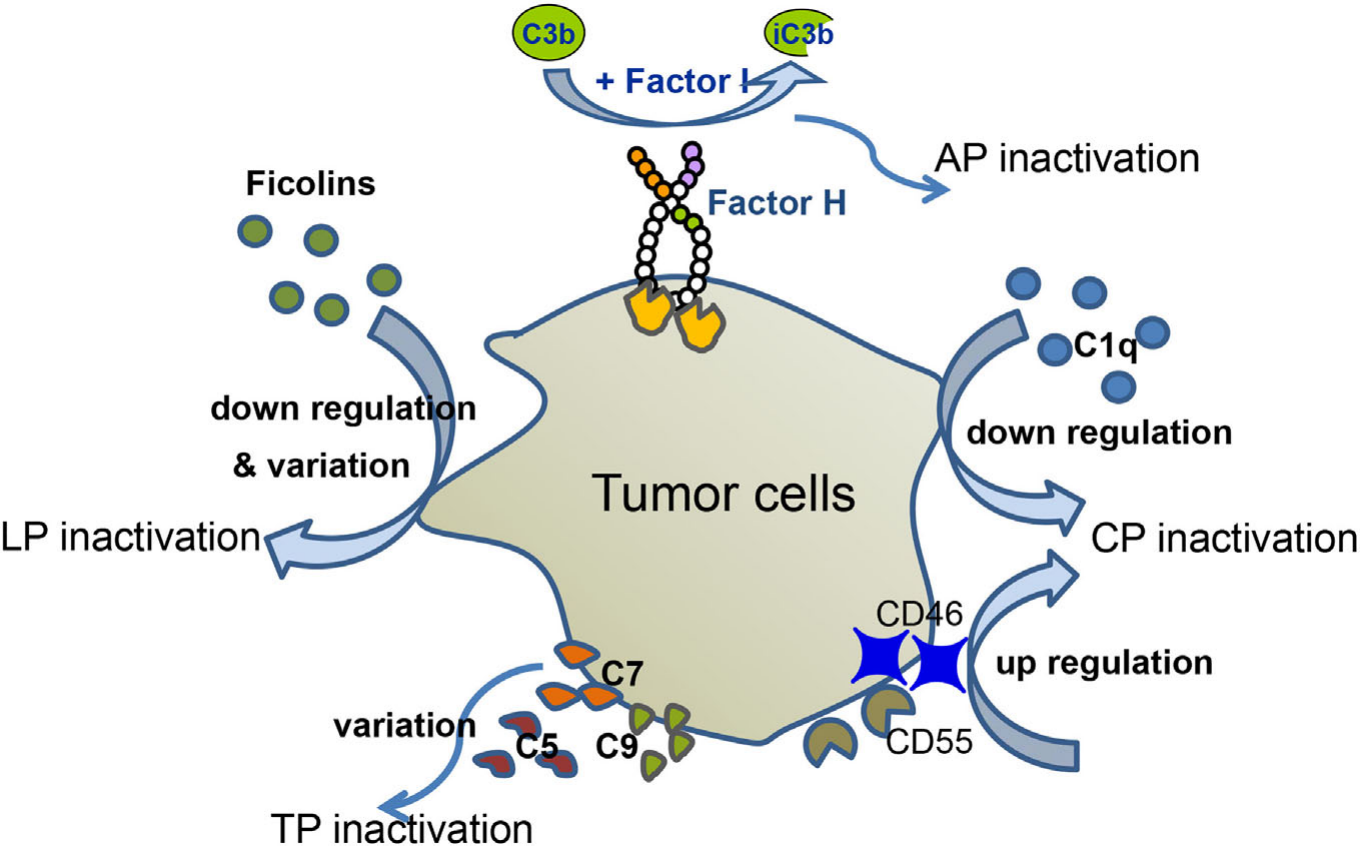
Complement evasion of hematological malignancies. Different types of hematological malignancies utilize different strategies for complement evasion. In MM patients, serum levels of C1q are down-regulated, which mediates CP inactivation. MM cells recruit Factor H to their surface, thereby down-regulating AP activation. Up-regulation of CD46 and CD55 inhibits complement activation. Down-regulation and variation of ficolin1, 2 lead to LP inactivation. In CLL patients, tumor cells altered C5 pattern to modify CP activation. Variation of C9 correlates with EFS of FL, and variation of C7 correlates with EFS of DLBCL.

The number of patients with hematological malignancies is increasing on a daily basis. Many patients with hematologic malignancies display abnormal levels of complement components, possibly as a result of the tumor avoiding complement surveillance (**Figure 2**). In this part, we will summarize the role of complement in hematological malignancy conditions.

### Complement in Multiple Myeloma

MM is a hematological malignant tumor characterized by clonal proliferation of plasma cells that produce M protein, accompanied by various types of impaired immune function. C1q, being one of the main components in CP, seems to be a potential marker of the immunodeficiency status in MM patients. Statistically, the mean serum level of C1q in MM patients is lower than that in the control group. At different stages of MM, C1q level also changes dynamically. When the disease is in remission, C1q is back to a normal level. However, with disease re-progressed, C1q level in the plasma decrease again, which indicates that MM cells, by controlling/consuming C1q, to some extent modulate the CP activation (49). Further, MM cells recruit complement regulator Factor H to their surface. Surface bound Factor H mediates factor I to cleave C3b, thereby down-regulating complement activation. Consistent with the above findings regarding complement deficiency, evidence shows that MM patients are at high risk of bacterial infections. No matter with normal or elevated level of C3, all sera from MM patients have a defect in C3b binding to *Streptococcus penumoniae.* Experimental data showed that addition of normal human serum to serum from MM patients restored the C3b binding ability to *S. pneumoniae*. However, adding anti-*S. pneumoniae* antibodies to MM serum did not rescue C3b deposition to any *S. pneumoniae* types. Such a scenario indicates that serum from MM patients has a defect in C3 activation, also explains why the MM patients have increased susceptibility to *S. pneumoniae* infections (50). Further, a recent clinical investigation showed that serumC3 and C4levels in MM patients were significantly higher than those in healthy people. Among the MM patients, serum C3 and C4 levels positively correlated with diseases severity. However, the mechanism how serum C3 and C4 promote MM development is not yet clear (51).

Ficolins (Ficolin 1, 2, 3) are complement lectin pathway defense factors, which are able to distinguish between “self”, “abnormal self” and “non-self”, thereby contributing to the elimination of “abnormal self” and “non-self’ by direct opsonization and/or initiation of complement activation through the lectin pathway. Lower level of ficolin-1 and ficolin-2 as well as polymorphisms of *FCN1* and *FCN2* genes were detected in patients diagnosed as MM, compared with control group, which leads to the higher hospital infections because of limited complement activation (52). The same group reported that in the early time a higher frequency of mannose-binding lectin deficiency-associated genotypes among MM patients was detected compared with controls (53).

### Complement in Leukemia

Leukemia is another type of life-threatening hematological malignancy. Experimental data show that leukemia cell lines together with clonogenic blasts from both CLL and acute myeloid leukemia (AML) patients respond strongly to C3 and C5 cleavage fragments in form of chemotaxis and increased adhesion. C3aR and C5aR were also detected at the mRNA and protein level in both human malignant hematopoietic cell lines and patient blasts upon stimulation by C3a and C5a (54). Further, abnormal C5 pattern approved in 42% CLL patients since C5 protein was detected as double bands accompanied by a lower molecular band when analyzed by Western blot, indicating that C5 cleavage/C5 activation happened, whereas C5 from the healthy controls and other CLL patients showed only one normal band (55). In comparison to patients with normal C5 pattern, patients with abnormal C5 pattern have increased basal levels of sC5b-9 and C5a, while sC5b-9 levels after CP activation were significantly decreased by 48%, suggesting CP activation was impaired. Such a scenario suggests a link between the pattern of C5 and CP activation (55) in CLL patients. In addition, a defect of C3b deposition on bacterial surface was detected although the serum concentrations of C3 are normal in patients with CLL. Mixing normal serum with serum from CLL patients restored C3b binding to bacterial surface, suggesting a defect in either the activation or activity of C3 in CLL serum, which likely accounts for the increased incidence of infections in these patients (56). Further, significant decrease of serum C1 and C4 levels was found in CLL patients, which correlated with the abnormal hemolytic activities. Meanwhile, a complement profile characteristic of acquired C1-IHN deficiency was observed in all tested patients, which indicates that the depression of the CP activity is a frequently occurring feature in CLL patients (57).

Moreover, different patterns of complement proteins were observed in different types of leukemia. In AML patients, each complement parameter tested was elevated as compared to the control values (sera of healthy blood donors). The extent of C3 activation and C3 splitting were correlated with disease severity while immunoglobin level remained consistently high and not varied much between different types of leukemia. Similar results were observed in acute lymphocytic leukemia patients although the differences were less marked. In chronic myelocytic leukemia, the CP and AP activities behave differently: activities of CP and serum C4 levels were significantly elevated, whereas activity of AP as well as serum C3 and factor B concentration were not significantly different from the control groups (58).

CR1 is expressed by erythrocytes and most leukocytes. sCR1, a soluble form, is shed from the leukocytes and found in plasma, which is identified as a powerful inhibitor of complement. Sadallah et al. reported sCR1 in the plasma of leukemia patients increased up to levels producing measurable complement inhibition, which is a possible complement evasion strategy utilized by leukemia cells (59).

### Complement in Non-Hodgkin Lymphoma

The most common types of NHL are DLBCL and FL. Germline mutations in complement genes have been associated with susceptibility to infections and autoimmune diseases, conditions that are associated with NHL risk (60). For example, genetic variations of complement genes (*e.g.*, MASP2, C5 and C9) are strongly associated with the development of NHL (60, 61). Charbonneau et al. also found that variations in C9 and complement regulatory genes (*e.g.*, Factor H, CFHR1, CFHR5, CD46, and CD55) were associated with the event-free survival (EFS) of FL, while C7 variations were associated with EFS of DLBCL (62). Thus, patients with different genotypes of complement components respond differently to complement attack and to antibody-based immunotherapy. Factor H, CFHR1 and CFHR5 polymorphisms have a stronger impact on EFS of FL patients who were treated with anti-CD20 antibody, while CD46 and CD55 polymorphisms had a stronger impact on EFS in FL patients who did not receive initial treatment. In addition, C1qA polymorphism seems to be a biomarker for predicting first-line response to R-CHOP regimen in DLBCL patients. In a retrospective analysis, DLBCL patients with C1qA homozygote A allele showed higher complete remission rates and longer overall survival after receiving R-CHOP regimen with an unknown mechanism (63). Further, over-expression of complement regulators, such as CD46, CD55 and CD59 is one of the main strategies utilized by NHL cell lines for complement evasion (64). All these data suggest a potential role of the complement system in NHL progression.

### The Co-Expression of Complement Genes by Hematological Malignancies and Solid Tumors

Many complement genes are co-expressed by different types of tumors. Roumenina et al. reported that there is strong heterogeneity in expression among different complement genes; however, not much difference existed between cancer types when comparing the transcription levels of 50 complement related genes within 30 types of tumors (mainly solid tumors) (65). When we further compared the co-expression profile of complement genes between hematological malignancies and solid tumors using the available data, both DLBCL and AML have higher expression level of complement *CFD* gene. Moreover, similar to most of the solid tumors, DLBCL also shows higher expression of *C3*, *ITGB2*, as well as genes of the classical pathway (*i.e.* *C1QA*, *C1QB*, *C1QC*, *C1R*, *C1S*, and *C2*), while the *C4BP*, lectin pathway genes (*i.e.* *MBL2, FCN*, and *MASP2*), and the terminal pathway genes (*i.e* *C6*, *C8.* and *C9*) are poorly expressed. However, different transcription profiles of the highly expressed genes are observed for the AML. Besides *CFD*, *FCN1, C1RL, C3AR1*, and *C5AR1* are strongly expressed, the rests including *C3* are all poorly expressed (**Supplementary Figure 1**, **Supplementary Table 1**). In addition, Roumenina L. and colleagues suggested that the co-expression of complement genes by the tumors confers poor or favorable prognosis or had no impact depending on the cancer type. In this scenario, both DLBC and AML fall in the group of “no impact on the prognosis” based on our further analysis (**Supplementary Figure 2**, **Supplementary Table 2**).

## Antibody-Based Immunotherapy Against Hematological Malignancies

### Different Types of Immunotherapies for the Treatment of Hematological Malignancies

Immunotherapy is becoming the mainstream for many types of hematological. malignancies. Hematological malignancies are derived from immune cells and are “sitting” in immune microenvironment, thereby having many opportunities to interact with resident immune cells, local antibodies, or complement system. This provides unique opportunities for immunotherapy. Currently, immunotherapy against hematological malignancies involves two major approaches: T cell therapies and antibody therapies. For T cell therapies, chimeric antigen receptor T (CAR-T) cell therapy is particularly promising for hematologic malignancies, garnering two FDA approvals using autologous cells in 2017 (66, 67), one for the treatment of pediatric ALL and the other for adult patients with advanced lymphomas (68). The main point for CAR-T therapy is generating engineered T cell by transfection of mRNA for CAR domain expression which typically consists an antigen-binding domain, a hinge that connects the scFv to a transmembrane domain and a signaling domain composed of CD3*ζ*. Antibody therapies include mAb and bispecific antibody. Blockade of PD-L1/PD-1 interaction has brought about another advance in immunotherapy for hematological malignancies. The clinical outcomes of anti-PD-1 mAbs on Hodgkin’s lymphoma are particularly impressive (69, 70). Combining mAb and CAR-T cell therapy, bispecific antibodies (BsAbs) were further developed, which contains one tumor antigen binding side and another side for binding and activating T cells. Due to the structure specificity, BsAbs can bridge T cells and target cells, thereby redirecting and activating T cell at sites of tumor cells (71). Currently, FBTA05 (Lymphomun) is a heterodimeric BsAb that recognizes CD20 and CD3, which has been used as monotherapy or followed by donor lymphocyte infusion in the treatment of CLL, high grade NHL, ALL, post-transplant lympho- proliferative disease. In addition, CD123- and CD33-specific BsAbs have been evaluated in clinical trials for patients with AML (72).

### Current Existing Monoclonal Antibodies Against Hematological Malignancies

Over the past two decades, the use of mAb and molecules derived from them has achieved considerable attention and success, establishing this mode of therapy as important therapeutic strategy in many cancers, especially in hematological malignancies. Among mAbs used for the treatment of hematological malignancies, anti-CD20 is the most routinely used and well characterized mAb for the treatment of the CD20-positive NHL and chronic CLL with major therapeutic advances (73). RTX, ofatumumab, ublituximab, veltuzumab, ocaratuzumab as well as tositumomab, obinutuzumab are currently available types. In addition, 97% of patients with classical HL typically exhibit an overexpression of PD-L1 due to the alteration in chromosome 9p24.1 (74). Therefore, the PD-1/PD-L1 axis is a good target for mAbs to kill tumor cells in HL. Nivolumab, a human IgG4 mAb, blocks the interaction of PD-L1 and PD-L2 by binding to the PD-1 receptor on activated immune cells, which was already approved by the FDA in 2016 for the treatment of relapsed or progressed HL (75). The anti-CD33 (*e.g*. immunotoxin, gemtuzumab, and ozogamicin) and anti-CD52 mAbs (alemtuzumab) are approved for treatment of relapsed AML in older patients and B-cell CLL. IGN523, targeting CD90 on the surface of malignant hematological cells (*e.g.* AML) is currently being evaluated in a Phase I clinical trial for AML (76). In addition, monoclonal antibodies targeting CD4, CD19, CD20, CD22, CD23, CD25, CD45, CD66, and CD122 are also under investigation in the clinic for the treatment of leukemia (77).

Breakthrough has also been made in targeting surface molecules expressed by MM cells, such as daratumumab, isatuximab, MOR202 as well as SAR650984 (different generations of humanized anti-CD38 monoclonal antibodies) (78), and elotuzumab, a humanized anti-signaling lymphocytic activation molecule family member 7 mAb. Among these mAbs, daratumumab and elotuzumab have been approved in the treatment of relapsed or refractory MM patients who received at least three prior therapies including proteasome inhibitors and immunomodulatory drugs (79). Investigational mAbs targeting CD138, CD56, CD40, CD74, BAFF, BCMA, GRP78, IGF-1R, and ICAM-1 on the surface of MM cells are pre-clinically developed, and several of them are in clinical trials (80).

### Effect of Complement on mAb-Based Immunotherapies

CDC and CDCP display a vital role in mAb-based immunotherapies (81). Approved for the treatment of hematological malignancies, most mAbs make use of complement in their mechanism of action. Upon application of mAbs, the complement pathways need to be fully effective to achieve better clinic efficacy. However, complement deficiencies, the over-expression of membrane complement regulatory proteins (*e.g.* CD55, CD59, and CD46) and fluid phase inhibitors (*e.g.* CFH, CFHR5, and C4BP) in the tumor microenvironment often cause resistance and non-responsiveness to mAb treatment (64, 82–84).

Di Gaetano et al. reported that the C1q-deficient mice exhibited a defective response to RTX in a lymphoma tumor mouse model. The work delineates the importance of CDC as an important effector mechanism in immunotherapy instead of ADCC as such an effect was not detected after depletion of either natural killer cells or granulocyte cells (85). Further, CLL patients undergoing complement deficiencies are suspected of limiting anti-CD20 mAb efficacy *in vivo* (83). In CLL, upon administration of RTX or ofatumumab, complement is quickly consumed (86, 87). C1 and C4 levels were below normal in more than 50% of the sera tested from CLL patients (57). Only when concurrent administration of fresh frozen plasma that RTX therapeutic activity can be restored in CLL patients. Polymorphisms of C1qA, C5, and C9 were often detected in FL and DLBCL patients, which may also limit complement activation, thereby affecting the clinical response to RTX (88).

Furthermore, complement regulators (CD55, CD59 and Factor H) limited RTX efficacy in CLL patients *via* down-regulating CDC (89). Functional block of these regulators significantly increased the susceptibility of primary CLL cells to anti-CD20 mAb. In FL and DLBCL patients, CD46, CD55, CD59, CFH, CFHR1, and CFHR5 gene expression likely affects the clinical response and duration of response to RTX therapy (90). Like CLL and NHL, MM cells also utilize complement regulators (CD55, CD59, and CFH) to block CDC, thereby resisting anti-CD38 mediated-antitumor immunotherapy (91). The serum levels of serglycin are elevated in patients with MM compared to healthy controls. Upon mAb treatment, serglycin protects MM cells from complement attack by blocking CDC, thereby promoting survival of malignant cells (32). Due to the different expression levels of each regulator/inhibitor of individual patients, mAb efficacy varies from patient to patient.

All in all, these comprehensive investigations that were performed *in vitro* studies and in mouse models as well as analyses from clinical patients provide key insights that functional complement is important for the mAb-based anti-tumor effects on hematological malignancies (62, 63). Down-regulation or inhibition of complement activation by hematological tumors is a big challenge for mAb-based immunotherapy.

### Improvement of Clinical Efficiency of mAb-Based Immunotherapies

To improve the clinical efficiency of mAbs, new strategies should be developed to initiate efficient complement activation by (1) mAb modifications (such as changing the target epitopes, Fc mutation, and immunoglobulin G subclass switching), (2) the control of membrane and soluble complement inhibitors, and (3) the concurrent administration of fresh frozen plasma during mAb therapy (84, 92).

Despite the clinical success achieved with RTX, incomplete treatment responses and emergence of resistance represent important limitations, suggesting further improvements of anti-CD20 mAb efficacy are required. Many novel anti-CD20 antibodies are under development either by changing the target CD20 epitope or by altering the Fc region to enhance immune effector cell activity (ADCC, ADCP). For example, compared with RTX, ofatumumab exhibits increased CDC by binding to a different CD20 epitope (93), while by Fc alterations, obinutuzumab has increased ADCC, reduced CDC, and enhanced direct non-apoptotic cell death (94, 95). CDC induction by obinutuzumab is 10 to 100-fold less than by the RTX and ofatumumab (93), resulting in a further-increased capacity to bind and activate natural killer cells in the presence of complement (96). In fact, Fc region engineering includes modifying the amino acid sequence or the glycosylation pattern, which allows enhancing both CDC and ADCC effector functions. Using this technique, four variants of RTX were generated as a native IgG1, a variant carrying the EFTAE modification (S267E/H268F/S324T/G236A/I332E) for enhanced CDC as well as glyco-engineered, non-fucosylated derivatives of both to boost ADCC. Antibodies with EFTAE modification were more efficacious in inducing CDC than antibodies with wild-type sequences due to enhanced C1q binding. Meanwhile, non-fucosylated variants had an enhanced affinity to Fc*γ*RIIIA and improved ADCC activity (97).

So far several types of anti-CD20 mAbs have been developed, such as RTX, ofatumumab (OFA), ublituximab, veltuzumab, ocaratuzumab as well as tositumomab and obinutuzumab. These variant anti-CD20 mAbs display different clinical efficiency because of CD20-binding characteristics and ability to induce CDC as well as ADCC (94). Structural analysis of CD20 by Rougé L. et al. nicely explains why some anti-CD20s are more efficient complement activators than others. The authors show that CD20 exists as a compact double-barrel dimer, which can be bound by two RTX antigen-binding fragments (Fabs). Each of the dimerized CD20 consists two parts, one as a composite epitope and the other one as an extensive homotypic Fab: Fab interface. RTX, by cross-linking CD20 into circular assemblies forms a structural model for complement recruitment, thereby leading to stronger complement activation (98). Kumar A. and his colleagues further used the cryo-electron microscopy to identify structures of full-length CD20 complexed either with prototypical type I (RTX and OFA) or type II (Obinutuzumab) mAbs. Their data showed that type I complexes function as molecular seeds to increase local concentration of mAbs, thereby effectively activating complement upon binding to CD20, which is similar to what Rougé L. et al. reported. However, type II complexes are unable to recruit additional mAbs and complement components, thereby failing to cause efficient complement activation. In addition, compared to RTX, OFA activates complement more potently because of the sharper binding angle of Fab_OFA_, suggesting that concatenation of IgG_OFA_ seeding complexes may bring their Fc domains in closer proximity, further facilitating their oligomerization (99).

Human IgG3 activates complement most efficiently among the IgG subclasses. An IgG3 switch variant of RTX induced better CDC even on low CD20 expressing cells compared with its parental IgG1 counterpart (100). Further, the shorter serum half-life of IgG3 can be rescued by the introduction of the R435H mutation, resulting in a potent mAb for CDC (101). IgG1/IgG3 chimera targeting CD20 showed stronger C1q binding, increased CDC capacity and more efficient B cell depletion in cynomolgus monkeys compared with the isotype matched parental mAbs (102, 103).

Furthermore, application of mAb together with inhibitors to mCRP as an adjuvant can achieve higher efficiency. The ongoing clinical trials of complement-related therapies for treatment of hematological malignancies are summarized in **Table 1**. Recombinant ILYd4, a novel CD59 inhibitor, effectively enhances the RTX-mediated CDC effect on RTX-sensitive RL-7 lymphoma cells and RTX-induced resistant RR51.2 cells. Meanwhile, recombinant ILYd4 also enhances the effect of RTX and anti-CD24 mAb on the refractory MM cell line ARH-77 (104, 105). Similarly, sorafenib potentiates RTX and ofatumumab efficacy in CLL and HL patients by decreasing the expression of complement regulatory proteins. Such effect of sorafenib has been investigated in more than 500 clinical trials with promising activity and good patients’ tolerance (110). Alternatively, mCRP function could be blocked by specific “neutralizing” antibodies. Blockage of CD55 and to a lesser extent of CD59 with specific antibodies *in vitro* significantly increased CDC of B lymphoma cells by RTX (111). However, the use of intact anti-mCRP antibodies *in vivo* may lead to CDC on healthy host cells. Mini-antibodies (MB-55, MB-59), composed of single-chain variable fragments to CD55 and CD59 and the human hinge-CH2–CH3 domains of IgG1, did not induce CDC themselves, but increased RTX mediated CDC by twofold *in vitro* (108). Their application in an *in vivo* model of human CD20 positive B cell lymphoma in SCID mice markedly increased survival upon RTX treatment (102).

**Table 1:** Complement-related therapies for treatment of hematological malignancies.

Therapies	Functions	Targeted molecules	Molecular nature	References
rILYd4	blocking CD59 regulatory function	CD59	30 amino acid fragments	(104, 105)
hSCR18-20	blocking Factor H regulatory function	Factor H	recombinant protein	(106, 107)
MB-59	blocking CD59 regulatory function	CD59	mini antibody	(108)
MB-55	blocking CD55 regulatory function	CD55	mini antibody	(108)
mAb A247	blocking Factor I function	Factor I	neutrolize antibody	(102, 109)
Sorafenib	decreasing the expression of complement regulatory Proteins	unknown	an oral compound	(110)
ATRA	decreasing the expression of complement regulatory proteins	unknown	Metabolic intermediates of vitamin A	(91)

ATRA, all-trans retinoic acid.

Moreover, neutralization of sCRPs is another way to enhance CDC. In combination with ofatumumab or RTX, human recombinant Factor H-derived short-consensus repeat 18–20 (hSCR18–20) increased susceptibility of primary CLL cells to CDC by abrogating Factor H function on the surface of CLL cells (106, 107). The decay of C3b to iC3b is strongly mediated by Factor I, for which most of the described CRPs exhibit cofactor function. Application of RTX or ofatumumab together with a neutralizing mAb against Factor I (mAb A247) increased CDC on CD20-expressing cell lines and primary CLL samples (102, 109). In addition, all-trans retinoic acid was reported to up-regulate CD38 expression level and down-regulate CD55 and CD59 level in daratumab-resistant MM cells, thereby enhancing the CDC effect on MM cells (91).

A clinical investigation by Xu et al. showed that application of fresh frozen plasma together with RTX is an effective measure to regain CDC effect upon the treatment of fludarabine refractory CLL patients. Twenty-two patients were treated with two units of fresh frozen plasma followed with RTX as a single agent, repeated every 1–2 weeks with a total of four courses of the combined fresh frozen plasma and RTX treatment. Sixteen patients (72.7%) responded to treatment, and seven (31.8%) achieved a complete remission. Three (13.6%) of them had no evidence of minimal residual disease after treatment (112). Such a clinical investigation suggests that the concurrent administration of fresh frozen plasma during mAb therapy is an optional measure to increase the mAb-induced clinical efficacy.

## Conclusions

This overview not only serves a fundamental understanding of the roles of complement in tumor progression and in mAb-based immunotherapy, but importantly, also highlights potential therapeutic targets/measures to improve the clinical efficacy of mAbs against hematological malignancies and further extrapolates this knowledge to other tumor related diseases. However, the mechanisms how complement affects hematological malignancies’ development and which strategy increases mAbs’ efficacy most clinically relevant are still elusive and need further investigation.

## Author Contributions

SL designed the outline of the manuscript, wrote, and reviewed the manuscript. MW wrote and reviewed the manuscript. HW revised the manuscript. DH wrote and reviewed the manuscript. YH and PZ discussed the topic and outlines of the manuscript and reviewed the text. All authors contributed to the article and approved the submitted version.

## Funding

This work was supported by the National Natural Science Foundations of China (No. 81601747, 82070136 to SL and No. 31770983, 81974249 to DH).

## Conflict of Interest

The authors declare that the research was conducted in the absence of any commercial or financial relationships that could be construed as a potential conflict of interest.
