# Supplementary material for: How Does Complement Affect Hematological Malignancies: From Basic Mechanisms to Clinical Application
Source: Front Immunol. 2020 Oct 29;11:593610. doi: 10.3389/fimmu.2020.593610 (PMC7658260; doi:10.3389/fimmu.2020.593610)
Supplement: Supplementary file 1 [file DataSheet_1.docx]

**Supplementary information**


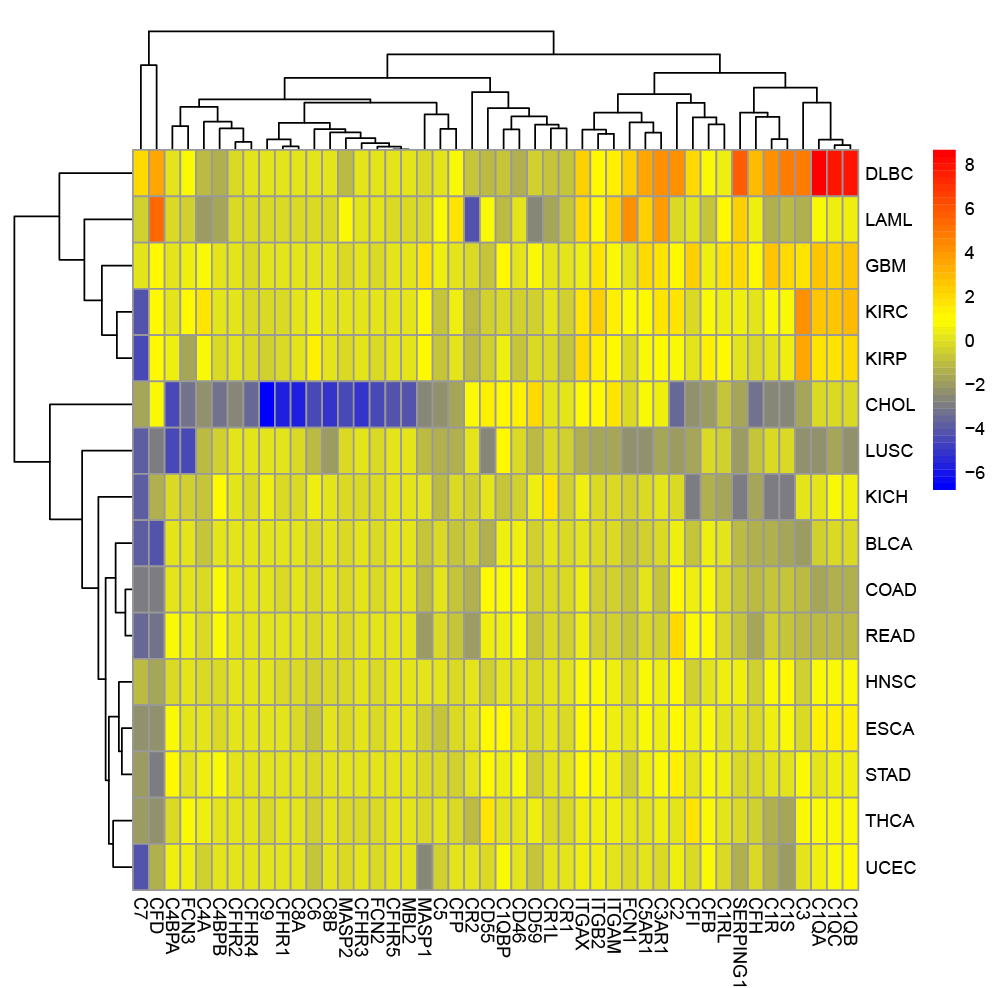


**Supplementary Figure 1.** Expression of complement genes in human cancers. The RNA-seq FPKM (Fragments Per Kilobase of transcript per Million Fragments) standardized expression data to evaluate complement transcriptome difference between different tumor samples and corresponding normal tissues was obtained through the UCSC Xena platform and come from The Cancer Genome Atlas (TCGA), and the data of normal tissues of LAML and DLBC was obtained from the Genotype-Tissue Expression (GTEx) project. Sixteen tumor types were used in this analysis. All analyses were performed using R software, the heatmap shows the expression of complement genes in different cancer types. Blue colors correspond to low expression, and red and yellow colors to high expression. Compared with other solid tumors, DLBCL and AML have stronger expression of complement CFD gene. Moreover, DLBCL is more strongly expressed on the genes of the classical pathway components (C1QA, C1QB, C1QC, C1R, C1S, C2). However, AML has weak complement gene expression in components of the classical pathway. BLCA, bladder carcinoma; C1QBP, C1q subcomponent- binding protein; C1RL, C1r subcomponent- like protein; C3AR1, C3a receptor 1; C4BP, C4b- binding protein; C5AR1, C5a receptor 1; CESC, cervical squamous carcinoma; CFHR, complement factor H-related protein; CHOL, cholangiocarcinoma; COAD, colon adenocarcinoma; CR , complement receptor; DLBC, diffuse large B cell lymphoma; ESCA, esophageal carcinoma; FCN, ficolin; GBM, glioblastoma; HNSC, head and neck squamous cell carcinoma; ITG, integrin; KICH, kidney chromophobe; KIRC, kidney renal clear cell carcinoma; KIRP, kidney renal papillary cell carcinoma; LAML, Acute Myeloid Leukemia; LUSC, lung squamous carcinoma; MASP, mannose- binding lectin- associated serine protease; MBL2, mannose- binding lectin 2; READ, rectum adenocarcinoma; STAD, stomach adenocarcinoma; THCA, thyroid carcinoma; UCEC, uterine corpus endometrial carcinoma.


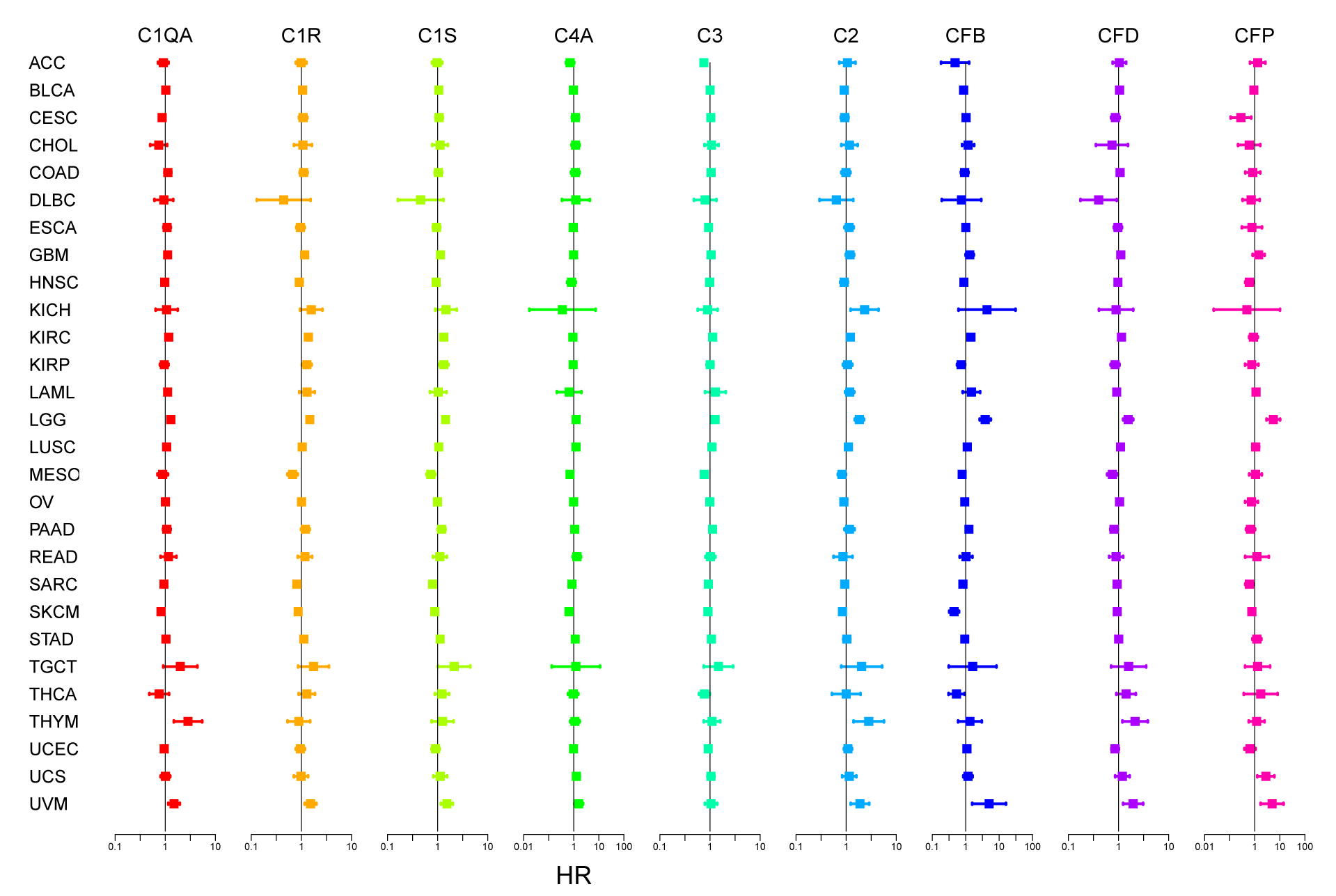


**Supplementary Figure 2.** Prognostic Impact of complement genes on the survival of patients with cancer. Multivariable Cox proportional hazards regression was used for analysis. ACC, adrenocortical carcinoma; BLCA, bladder carcinoma; CESC, cervical squamous carcinoma; CHOL, cholangiocarcinoma; COAD, colon adenocarcinoma; CI, confidence interval;DLBC, diffuse large B cell lymphoma; ESCA, esophageal carcinoma;GBM, glioblastoma; HNSC, head and neck squamous cell carcinoma; HR, hazard ratio; KICH, kidney chromophobe; KIRC, kidney renal clear cell carcinoma; KIRP, kidney renal papillary cell carcinoma; LAML, Acute Myeloid Leukemia; LGG, lower grade glioma; LUSC, lung squamous carcinoma; MESO, mesothelioma; OV, ovarian serous cystadenocarcinoma; PAAD, pancreatic adenocarcinoma; READ, rectum adenocarcinoma;SARC, sarcoma; SKCM, skin cutaneous melanoma; STAD, stomach adenocarcinoma; TGCT, testicular germ cell tumor; THCA, thyroid carcinoma; THYM, thymoma; UCEC, uterine corpus endometrial carcinoma; UCS, uterine carcinosarcoma; UVM, uveal melanoma.

**Supplementary Table 1. The *P* value of the differential expression of complement genes in human cancers (compared with corresponding normal tissues)**

|  | Cancer Type | | | | | | | | | | | | | | | |
| --- | --- | --- | --- | --- | --- | --- | --- | --- | --- | --- | --- | --- | --- | --- | --- | --- |
| Gene | BLCA | CHOL | COAD | DLBC | ESCA | GBM | HNSC | KICH | KIRC | KIRP | LAML | LUSC | READ | STAD | THCA | UCEC |
| CD59 | 0.003 | 0.000 | 0.000 | 0.000 | 0.192 | 0.015 | 0.232 | 0.006 | 0.000 | 0.028 | 0.000 | 0.000 | 0.000 | 0.001 | 0.000 | 0.000 |
| C3 | 0.000 | 0.000 | 0.000 | 0.000 | 0.879 | 0.002 | 0.089 | 0.334 | 0.000 | 0.000 | 0.000 | 0.000 | 0.017 | 0.013 | 0.000 | 0.537 |
| C4A | 0.000 | 0.000 | 0.002 | 0.000 | 0.552 | 0.050 | 0.832 | 0.000 | 0.000 | 0.002 | 0.000 | 0.000 | 0.393 | 0.000 | 0.000 | 0.000 |
| C1QBP | 0.005 | 0.000 | 0.000 | 0.000 | 0.000 | 0.000 | 0.018 | 0.000 | 0.000 | 0.000 | 0.000 | 0.000 | 0.027 | 0.000 | 0.706 | 0.000 |
| ITGB2 | 0.613 | 0.028 | 0.001 | 0.000 | 0.002 | 0.001 | 0.000 | 0.435 | 0.000 | 0.000 | 0.000 | 0.000 | 0.055 | 0.001 | 0.005 | 0.005 |
| C1QC | 0.780 | 0.944 | 0.000 | 0.000 | 0.003 | 0.000 | 0.000 | 0.036 | 0.000 | 0.000 | 0.204 | 0.000 | 0.007 | 0.061 | 0.000 | 0.000 |
| C1QA | 0.175 | 0.770 | 0.000 | 0.000 | 0.009 | 0.000 | 0.000 | 0.290 | 0.000 | 0.000 | 0.232 | 0.000 | 0.000 | 0.476 | 0.000 | 0.017 |
| C1QB | 0.563 | 0.727 | 0.000 | 0.000 | 0.004 | 0.000 | 0.000 | 0.043 | 0.000 | 0.000 | 0.023 | 0.000 | 0.011 | 0.087 | 0.000 | 0.000 |
| CD46 | 0.012 | 0.000 | 0.000 | 0.000 | 0.188 | 0.275 | 0.897 | 0.000 | 0.000 | 0.000 | 0.012 | 0.006 | 0.000 | 0.000 | 0.000 | 0.726 |
| SERPING1 | 0.001 | 0.000 | 0.000 | 0.000 | 0.340 | 0.000 | 0.004 | 0.000 | 0.000 | 0.078 | 0.000 | 0.000 | 0.011 | 0.828 | 0.202 | 0.000 |
| C1R | 0.000 | 0.000 | 0.000 | 0.000 | 0.062 | 0.000 | 0.000 | 0.000 | 0.000 | 0.549 | 0.000 | 0.273 | 0.060 | 0.379 | 0.000 | 0.000 |
| C1S | 0.000 | 0.000 | 0.000 | 0.000 | 0.083 | 0.001 | 0.000 | 0.000 | 0.000 | 0.050 | 0.000 | 0.408 | 0.005 | 0.370 | 0.000 | 0.000 |
| CFB | 0.073 | 0.000 | 0.000 | 0.000 | 0.069 | 0.072 | 0.078 | 0.000 | 0.000 | 0.000 | 0.000 | 0.629 | 0.000 | 0.003 | 0.000 | 0.000 |
| CD55 | 0.001 | 0.000 | 0.000 | 0.000 | 0.023 | 0.012 | 0.036 | 0.654 | 0.000 | 0.000 | 0.000 | 0.000 | 0.024 | 0.000 | 0.000 | 0.588 |
| C2 | 0.201 | 0.000 | 0.000 | 0.000 | 0.000 | 0.001 | 0.000 | 0.945 | 0.000 | 0.005 | 0.000 | 0.000 | 0.000 | 0.000 | 0.000 | 0.005 |
| C1RL | 0.122 | 0.000 | 0.051 | 0.000 | 0.548 | 0.000 | 0.000 | 0.000 | 0.000 | 0.000 | 0.000 | 0.000 | 0.210 | 0.001 | 0.000 | 0.752 |
| CFH | 0.000 | 0.000 | 0.000 | 0.000 | 0.488 | 0.004 | 0.009 | 0.000 | 0.031 | 0.335 | 0.192 | 0.000 | 0.000 | 0.656 | 0.173 | 0.165 |
| CR1L | 0.004 | 0.418 | 0.005 | 0.000 | 0.095 | 0.250 | 0.000 | 0.000 | 0.000 | 0.000 | 0.000 | 0.002 | 0.074 | 0.000 | 0.002 | 0.000 |
| MASP2 | 0.038 | 0.000 | 0.000 | 0.000 | 0.005 | 0.080 | 0.238 | 0.000 | 0.000 | 0.032 | 0.000 | 0.000 | 0.022 | 0.187 | 0.000 | 0.000 |
| CFHR1 | 0.172 | 0.000 | 0.001 | 0.098 | 0.539 | 0.385 | 0.568 | 0.000 | 0.000 | 0.000 | 0.000 | 0.764 | 0.007 | 0.155 | 0.045 | 0.124 |
| CFHR3 | 0.688 | 0.000 | 0.000 | 0.000 | 0.101 | 0.193 | 0.000 | 0.000 | 0.593 | 0.011 | 0.005 | 0.000 | 0.002 | 0.004 | 0.008 | 0.455 |
| FCN2 | 0.000 | 0.000 | 0.000 | 0.192 | 0.003 | 0.016 | 0.000 | 0.010 | 0.510 | 0.000 | 0.000 | 0.000 | 0.164 | 0.011 | 0.000 | 0.000 |
| C9 | 0.052 | 0.000 | 0.007 | 0.177 | 0.013 | 0.398 | 0.000 | 0.000 | 0.000 | 0.000 | 0.177 | 0.000 | 0.473 | 0.000 | 0.000 | 0.166 |
| C8A | 0.651 | 0.000 | 0.018 | 0.000 | 0.580 | 0.276 | 0.933 | 0.002 | 0.078 | 0.278 | 0.000 | 0.000 | 0.146 | 0.500 | 0.000 | 0.183 |
| C8B | 0.065 | 0.000 | 0.373 | 0.002 | 0.188 | 0.099 | 0.643 | 0.002 | 0.000 | 0.000 | 0.000 | 0.000 | 0.117 | 0.353 | 0.000 | 0.380 |
| CFHR2 | 0.517 | 0.000 | 0.439 | 0.000 | 0.457 | 0.890 | 0.679 | 0.168 | 0.182 | 0.457 | 0.000 | 0.443 | 0.680 | 0.141 | 0.501 | 0.941 |
| CFHR5 | 0.547 | 0.000 | 0.640 | 0.000 | 0.395 | 0.243 | 0.130 | 0.221 | 0.001 | 0.041 | 0.000 | 0.176 | 0.327 | 0.006 | 0.628 | 0.068 |
| CFHR4 | 0.005 | 0.000 | 0.278 | 0.000 | 0.022 | 0.002 | 0.000 | 0.000 | 0.000 | 0.000 | 0.000 | 0.000 | 0.958 | 0.000 | 0.178 | 0.003 |
| MBL2 | 0.161 | 0.000 | 0.272 | 0.000 | 0.116 | 0.378 | 0.003 | 0.007 | 0.000 | 0.014 | 0.000 | 0.012 | 0.970 | 0.078 | 0.326 | 0.804 |
| C7 | 0.000 | 0.008 | 0.000 | 0.000 | 0.000 | 0.674 | 0.000 | 0.000 | 0.000 | 0.000 | 0.000 | 0.000 | 0.000 | 0.000 | 0.000 | 0.000 |
| CFI | 0.001 | 0.000 | 0.000 | 0.000 | 0.059 | 0.000 | 0.053 | 0.000 | 0.071 | 0.052 | 0.000 | 0.000 | 0.018 | 0.287 | 0.000 | 0.904 |
| C5 | 0.005 | 0.000 | 0.000 | 0.163 | 0.032 | 0.020 | 0.374 | 0.000 | 0.000 | 0.000 | 0.000 | 0.000 | 0.386 | 0.077 | 0.673 | 0.000 |
| ITGAX | 0.198 | 0.000 | 0.000 | 0.000 | 0.000 | 0.138 | 0.000 | 0.001 | 0.000 | 0.000 | 0.000 | 0.000 | 0.054 | 0.000 | 0.002 | 0.000 |
| CFD | 0.000 | 0.035 | 0.000 | 0.000 | 0.000 | 0.006 | 0.000 | 0.000 | 0.000 | 0.000 | 0.000 | 0.000 | 0.000 | 0.000 | 0.000 | 0.000 |
| C5AR1 | 0.015 | 0.002 | 0.776 | 0.000 | 0.004 | 0.001 | 0.000 | 0.792 | 0.000 | 0.000 | 0.000 | 0.000 | 0.511 | 0.007 | 0.190 | 0.467 |
| C3AR1 | 0.235 | 0.081 | 0.000 | 0.000 | 0.042 | 0.001 | 0.000 | 0.835 | 0.000 | 0.000 | 0.000 | 0.000 | 0.028 | 0.000 | 0.007 | 0.615 |
| ITGAM | 0.153 | 0.000 | 0.000 | 0.000 | 0.032 | 0.018 | 0.000 | 0.729 | 0.000 | 0.000 | 0.000 | 0.000 | 0.008 | 0.004 | 0.000 | 0.504 |
| C6 | 0.000 | 0.000 | 0.000 | 0.068 | 0.000 | 0.196 | 0.000 | 0.257 | 0.197 | 0.001 | 0.000 | 0.000 | 0.000 | 0.005 | 0.000 | 0.000 |
| MASP1 | 0.000 | 0.000 | 0.000 | 0.002 | 0.031 | 0.002 | 0.074 | 0.167 | 0.000 | 0.000 | 0.000 | 0.000 | 0.000 | 0.000 | 0.000 | 0.000 |
| C4BPA | 0.081 | 0.000 | 0.244 | 0.752 | 0.312 | 0.754 | 0.004 | 0.000 | 0.007 | 0.013 | 0.002 | 0.000 | 0.007 | 0.000 | 0.000 | 0.480 |
| C4BPB | 0.042 | 0.000 | 0.000 | 0.000 | 0.349 | 0.810 | 0.000 | 0.000 | 0.000 | 0.000 | 0.000 | 0.000 | 0.000 | 0.000 | 0.063 | 0.032 |
| CR2 | 0.273 | 0.012 | 0.000 | 0.062 | 0.763 | 0.000 | 0.600 | 0.071 | 0.000 | 0.000 | 0.000 | 0.199 | 0.000 | 0.466 | 0.000 | 0.000 |
| FCN3 | 0.013 | 0.000 | 0.000 | 0.000 | 0.231 | 0.001 | 0.000 | 0.316 | 0.000 | 0.000 | 0.000 | 0.000 | 0.000 | 0.000 | 0.000 | 0.001 |
| CFP | 0.000 | 0.000 | 0.000 | 0.003 | 0.988 | 0.317 | 0.000 | 0.008 | 0.000 | 0.024 | 0.000 | 0.000 | 0.000 | 0.000 | 0.219 | 0.960 |
| CR1 | 0.001 | 0.493 | 0.000 | 0.000 | 0.561 | 0.571 | 0.924 | 0.000 | 0.000 | 0.000 | 0.000 | 0.000 | 0.000 | 0.331 | 0.830 | 0.438 |
| FCN1 | 0.000 | 0.306 | 0.000 | 0.000 | 0.889 | 0.263 | 0.286 | 0.015 | 0.000 | 0.631 | 0.000 | 0.000 | 0.000 | 0.002 | 0.087 | 0.228 |

*P* < 0.05 shows statistical significance.

Abbreviations: BLCA, bladder carcinoma;C1QBP, C1q subcomponent- binding protein; C1RL, C1r subcomponent- like protein; *C3AR1*, C3a receptor 1; *C4BP*,C4b- binding protein; *C5AR1*, C5a receptor 1; CESC, cervical squamous carcinoma; *CFHR*, complement factor H-related protein; CHOL, cholangiocarcinoma; COAD, colon adenocarcinoma; CR , complement receptor; DLBC, diffuse large B cell lymphoma; ESCA, esophageal carcinoma; *FCN*, ficolin; GBM, glioblastoma; HNSC, head and neck squamous cell carcinoma; *ITG*, integrin; KICH, kidney chromophobe; KIRC, kidney renal clear cell carcinoma; KIRP, kidney renal papillary cell carcinoma; LAML, Acute Myeloid Leukemia; LUSC, lung squamous carcinoma; MASP, mannose- binding lectin- associated serine protease; *MBL2*, mannose- binding lectin 2; READ, rectum adenocarcinoma; STAD, stomach adenocarcinoma; THCA, thyroid carcinoma; UCEC, uterine corpus endometrial carcinoma.

**Supplementary Table 2. Prognostic Impact of complement genes on the survival of patients with cancer**

|  | Complement Gene | | | | | | | | | | | | | | | | | |
| --- | --- | --- | --- | --- | --- | --- | --- | --- | --- | --- | --- | --- | --- | --- | --- | --- | --- | --- |
|  | C1QA | | C1R | | C1S | | C4A | | C2 | | C3 | | CFB | | CFD | | CFP | |
| Cancer  Type | HR (95%CI) | *P* | HR (95%CI) | *P* | HR (95%CI) | *P* | HR (95%CI) | *P* | HR (95%CI) | *P* | HR (95%CI) | *P* | HR (95%CI) | *P* | HR (95%CI) | *P* | HR (95%CI) | *P* |
| ACC | 0.92  (0.72-1.16) | 0.468 | 0.99  (0.78-1.26) | 0.938 | 0.98  (0.77-1.24) | 0.857 | 0.71  (0.49-1.04) | 0.075 | 1.06  (0.73-1.53) | 0.766 | 0.75  (0.65-0.87) | *< 0.001 | 0.48  (0.18-1.27) | 0.139 | 1.04  (0.76-1.42) | 0.805 | 1.3  (0.64-2.66) | 0.463 |
| BLCA | 1.02  (0.95-1.1) | 0.529 | 1.05  (0.96-1.16) | 0.296 | 1.06  (0.97-1.15) | 0.202 | 0.96  (0.79-1.17) | 0.690 | 0.91  (0.81-1.03) | 0.131 | 1.01  (0.93-1.08) | 0.89 | 0.87  (0.75-1.01) | 0.061 | 1.05  (0.95-1.16) | 0.332 | 0.92  (0.71-1.19) | 0.517 |
| CESC | 0.87  (0.76-0.99) | *0.04 | 1.08  (0.89-1.31) | 0.424 | 1.07  (0.91-1.26) | 0.426 | 1.16  (0.84-1.59) | 0.372 | 0.93  (0.78-1.12) | 0.451 | 1.04  (0.91-1.18) | 0.57 | 1.03  (0.87-1.21) | 0.751 | 0.86  (0.71-1.05) | 0.139 | 0.28  (0.11-0.72) | *0.008 |
| CHOL | 0.74  (0.5-1.09) | 0.131 | 1.07  (0.71-1.62) | 0.740 | 1.12  (0.78-1.6) | 0.538 | 1.18  (0.82-1.71) | 0.378 | 1.16  (0.8-1.69) | 0.426 | 1.07  (0.78-1.48) | 0.67 | 1.18  (0.78-1.79) | 0.435 | 0.74  (0.35-1.54) | 0.418 | 0.6  (0.22-1.66) | 0.324 |
| COAD | 1.13  (0.97-1.32) | 0.123 | 1.11  (0.92-1.32) | 0.273 | 1.04  (0.88-1.23) | 0.648 | 1.15  (0.78-1.7) | 0.482 | 0.99  (0.8-1.21) | 0.906 | 1.05  (0.93-1.19) | 0.41 | 0.93 (0.72-1.21) | 0.603 | 1.07  (0.95-1.21) | 0.254 | 0.83  (0.42-1.66) | 0.604 |
| DLBC | 0.94  (0.61-1.44) | 0.771 | 0.44  (0.13-1.52) | 0.196 | 0.46  (0.16-1.31) | 0.144 | 1.21  (0.34-4.34) | 0.770 | 0.64  (0.29-1.38) | 0.254 | 0.8  (0.48-1.34) | 0.40 | 0.75  (0.19-2.92) | 0.679 | 0.4  (0.17-0.92) | *0.031 | 0.71  (0.33-1.56) | 0.399 |
| ESCA | 1.09  (0.92-1.29) | 0.320 | 0.96  (0.79-1.16) | 0.678 | 0.95  (0.81-1.13) | 0.563 | 0.95  (0.73-1.23) | 0.706 | 1.15  (0.93-1.42) | 0.205 | 0.94  (0.83-1.06) | 0.29 | 1.01  (0.85-1.2) | 0.919 | 0.97  (0.81-1.17) | 0.758 | 0.77  (0.31-1.93) | 0.575 |
| GBM | 1.12  (0.96-1.29) | 0.141 | 1.16 (1-1.34) | *0.047 | 1.14  (0.99-1.32) | 0.064 | 0.98  (0.81-1.18) | 0.833 | 1.19  (0.98-1.44) | 0.075 | 1.05  (0.91-1.22) | 0.52 | 1.32 (1-1.74) | 0.053 | 1.1  (0.96-1.26) | 0.151 | 1.43  (0.82-2.49) | 0.202 |
| HNSC | 0.98  (0.89-1.07) | 0.656 | 0.91  (0.78-1.05) | 0.205 | 0.94  (0.83-1.06) | 0.291 | 0.81  (0.54-1.2) | 0.290 | 0.91  (0.77-1.08) | 0.278 | 0.98  (0.9-1.08) | 0.74 | 0.88  (0.75-1.04) | 0.145 | 0.97  (0.86-1.09) | 0.594 | 0.61  (0.42-0.89) | *0.009 |
| KICH | 1.07  (0.64-1.77) | 0.800 | 1.57  (0.94-2.63) | 0.085 | 1.47  (0.9-2.41) | 0.127 | 0.35  (0.02-7.38) | 0.499 | 2.33  (1.23-4.42) | *0.010 | 0.9  (0.57-1.42) | 0.64 | 4.36  (0.61-31.09) | 0.142 | 0.89 (0.41-1.95) | 0.774 | 0.49  (0.02-10.16) | 0.642 |
| KIRC | 1.18  (1.03-1.35) | *0.018 | 1.37  (1.23-1.53) | *< 0.001 | 1.33  (1.19-1.48) | *< 0.001 | 0.92  (0.81-1.05) | 0.206 | 1.21  (1.09-1.35) | *0.001 | 1.13  (1.02-1.25) | *0.02 | 1.43  (1.21-1.69) | *< 0.001 | 1.14 (1-1.3) | *0.044 | 0.88  (0.6-1.32) | 0.545 |
| KIRP | 0.96  (0.79-1.16) | 0.660 | 1.28  (1.03-1.6) | *0.027 | 1.32  (1.07-1.63) | *0.008 | 0.94  (0.75-1.19) | 0.606 | 1.07  (0.86-1.33) | 0.556 | 1 (0.86-1.17) | 0.95 | 0.73  (0.55-0.96) | *0.025 | 0.85  (0.7-1.04) | 0.123 | 0.76  (0.41-1.4) | 0.375 |
| LAML | 1.11  (0.99-1.25) | 0.068 | 1.29  (0.91-1.85) | 0.155 | 1.03 (0.7-1.5) | 0.885 | 0.65  (0.21-2.01) | 0.455 | 1.17  (0.95-1.44) | 0.128 | 1.29  (0.8-2.07) | 0.30 | 1.49  (0.82-2.71) | 0.194 | 0.92  (0.83-1.01) | 0.067 | 1.12  (0.94-1.33) | 0.218 |
| LGG | 1.29  (1.12-1.48) | *< 0.001 | 1.47  (1.32-1.63) | *< 0.001 | 1.44  (1.28-1.61) | *< 0.001 | 1.23  (1.03-1.47) | *0.020 | 1.83  (1.49-2.27) | *< 0.001 | 1.26  (1.12-1.42) | *< 0.001 | 3.85  (2.63-5.64) | *< 0.001 | 1.55  (1.24-1.96) | *< 0.001 | 5.49  (2.95-10.23) | *< 0.001 |
| LUSC | 1.07  (0.96-1.18) | 0.211 | 1.04  (0.9-1.2) | 0.629 | 1.06  (0.94-1.19) | 0.369 | 1.23  (1.01-1.49) | *0.037 | 1.11  (0.96-1.28) | 0.151 | 1.09  (0.99-1.21) | 0.09 | 1.12  (0.96-1.3) | 0.147 | 1.09  (0.96-1.25) | 0.182 | 1.09  (0.8-1.49) | 0.599 |
| MESO | 0.89  (0.7-1.13) | 0.339 | 0.67  (0.53-0.84) | *0.001 | 0.73 (0.6-0.9) | *0.002 | 0.7  (0.59-0.84) | *< 0.001 | 0.82  (0.68-0.98) | *0.032 | 0.77  (0.67-0.88) | *< 0.001 | 0.78  (0.69-0.89) | *< 0.001 | 0.75  (0.59-0.96) | *0.023 | 1.07  (0.6-1.92) | 0.819 |
| OV | 1.01  (0.92-1.12) | 0.820 | 1.01  (0.89-1.15) | 0.900 | 0.99 (0.9-1.1) | 0.920 | 0.98  (0.89-1.08) | 0.691 | 0.9 (0.8-1) | 0.060 | 0.99  (0.93-1.06) | 0.85 | 0.94  (0.85-1.03) | 0.196 | 1.05  (0.92-1.19) | 0.483 | 0.74  (0.41-1.34) | 0.318 |
| PAAD | 1.08  (0.9-1.28) | 0.410 | 1.2  (0.99-1.46) | 0.064 | 1.2  (1.01-1.43) | 0.041 | 1.1  (0.84-1.43) | 0.495 | 1.16  (0.92-1.47) | 0.196 | 1.12  (0.99-1.27) | *0.07 | 1.25  (1.02-1.53) | *0.030 | 0.81  (0.68-0.96) | *0.016 | 0.68  (0.45-1.02) | *0.064 |
| READ | 1.16  (0.81-1.67) | 0.418 | 1.18  (0.84-1.64) | 0.340 | 1.11  (0.81-1.53) | 0.515 | 1.33  (0.91-1.94) | 0.139 | 0.86  (0.56-1.33) | 0.507 | 1.02  (0.81-1.28) | 0.88 | 1.03  (0.67-1.59) | 0.900 | 0.89  (0.65-1.23) | 0.492 | 1.22  (0.41-3.62) | 0.714 |
| SARC | 0.94  (0.86-1.03) | 0.213 | 0.81  (0.71-0.92) | *0.001 | 0.79  (0.71-0.88) | *< 0.001 | 0.84  (0.7-1.02) | 0.084 | 0.94  (0.83-1.06) | 0.308 | 0.93 (0.86-1) | 0.06 | 0.83  (0.68-1.01) | 0.061 | 0.94  (0.87-1.02) | 0.111 | 0.61  (0.42-0.88) | *0.008 |
| SKCM | 0.83  (0.77-0.89) | *0.000 | 0.86  (0.79-0.95) | 0.002 | 0.88  (0.81-0.96) | 0.003 | 0.65  (0.51-0.83) | 0.001 | 0.84  (0.77-0.92) | 0.000 | 0.91  (0.85-0.97) | 0.01 | 0.45  (0.32-0.63) | 0.000 | 0.94  (0.84-1.05) | 0.276 | 0.76  (0.61-0.95) | 0.016 |
| STAD | 1.03  (0.92-1.16) | 0.596 | 1.12  (0.96-1.3) | 0.140 | 1.13  (0.98-1.3) | 0.103 | 1.14  (0.96-1.35) | 0.124 | 1.03  (0.88-1.21) | 0.735 | 1.07  (0.97-1.18) | 0.17 | 0.94  (0.82-1.08) | 0.364 | 1 (0.9-1.13) | 0.943 | 1.21  (0.81-1.82) | 0.352 |
| TGCT | 2 (0.91-4.38) | 0.084 | 1.75  (0.86-3.55) | 0.123 | 2.14  (1.02-4.49) | 0.045 | 1.21  (0.13-11.14) | 0.865 | 2.04 (0.8-5.2) | 0.137 | 1.47  (0.75-2.88) | 0.26 | 1.61  (0.31-8.35) | 0.568 | 1.59  (0.71-3.55) | 0.259 | 1.29  (0.41-4.08) | 0.659 |
| THCA | 0.76  (0.48-1.19) | 0.230 | 1.28  (0.88-1.86) | 0.192 | 1.22  (0.88-1.69) | 0.227 | 0.94  (0.59-1.51) | 0.808 | 1 (0.52-1.93) | 0.993 | 0.77  (0.61-0.99) | 0.04 | 0.53  (0.31-0.92) | 0.024 | 1.41 (0.9-2.2) | 0.129 | 1.72  (0.36-8.11) | 0.493 |
| THYM | 2.84  (1.48-5.46) | *0.002 | 0.89  (0.53-1.49) | 0.652 | 1.26  (0.76-2.08) | 0.371 | 1.11  (0.72-1.72) | 0.636 | 2.82  (1.41-5.66) | 0.004 | 1.1 (0.75-1.6) | 0.63 | 1.34  (0.59-3.03) | 0.480 | 2.13  (1.19-3.83) | 0.011 | 1.19  (0.57-2.46) | 0.642 |
| UCEC | 0.96  (0.83-1.1) | 0.538 | 0.95  (0.78-1.18) | 0.661 | 0.91  (0.76-1.09) | 0.317 | 0.97  (0.75-1.27) | 0.848 | 1.08 (0.9-1.3) | 0.386 | 0.92  (0.84-1.01) | 0.08 | 1.09  (0.93-1.28) | 0.292 | 0.85  (0.72-1.01) | 0.067 | 0.65  (0.39-1.08) | 0.095 |
| UCS | 1.01  (0.81-1.25) | 0.930 | 0.98  (0.71-1.36) | 0.904 | 1.13  (0.82-1.55) | 0.457 | 1.27  (0.94-1.72) | 0.123 | 1.15  (0.83-1.59) | 0.400 | 1.05  (0.89-1.23) | 0.57 | 1.17  (0.85-1.61) | 0.328 | 1.19  (0.86-1.66) | 0.300 | 2.77  (1.26-6.09) | 0.011 |
| UVM | 1.5  (1.15-1.96) | *0.003 | 1.52  (1.16-1.99) | 0.002 | 1.53  (1.16-2.02) | 0.002 | 1.57  (1.06-2.33) | 0.024 | 1.88  (1.23-2.88) | 0.003 | 1.05  (0.79-1.39) | 0.75 | 5.02  (1.56-16.1) | 0.007 | 1.95  (1.23-3.08) | 0.004 | 4.92  (1.72-14.05) | 0.003 |

**p* < 0.05 shows statistical significance.

Abbreviations: ACC, adrenocortical carcinoma; BLCA, bladder carcinoma;CESC, cervical squamouscarcinoma; CHOL, cholangiocarcinoma; COAD, colon adenocarcinoma; CI, confidence interval; DLBC, diffuse large B cell lymphoma; ESCA, esophageal carcinoma; GBM, glioblastoma; HNSC, head and neck squamous cell carcinoma; HR, hazard ratio; KICH, kidney chromophobe; KIRC, kidney renal clear cell carcinoma; KIRP, kidney renal papillary cell carcinoma; LAML, Acute Myeloid Leukemia; LGG, lower grade glioma; LUSC, lung squamous carcinoma; MESO, mesothelioma; OV, ovarian serous cystadenocarcinoma; PAAD, pancreatic adenocarcinoma; READ, rectum adenocarcinoma; SARC, sarcoma; SKCM, skin cutaneous melanoma; STAD, stomach adenocarcinoma; TGCT, testicular germ cell tumor; THCA, thyroid carcinoma; THYM, thymoma; UCEC, uterine corpus endometrial carcinoma; UCS, uterine carcinosarcoma; UVM, uveal melanoma.
